# Genomic epidemiology of severe community-onset *Acinetobacter baumannii* infection

**DOI:** 10.1099/mgen.0.000258

**Published:** 2019-02-26

**Authors:** Ella M. Meumann, Nicholas M. Anstey, Bart J. Currie, Kim A. Piera, Johanna J. Kenyon, Ruth M. Hall, Joshua S. Davis, Derek S. Sarovich

**Affiliations:** ^1^​ Global and Tropical Health Division, Menzies School of Health Research, Darwin 0810, Australia; ^2^​ Department of Infectious Diseases, Royal Darwin Hospital, Darwin 0810, Australia; ^3^​ School of Biomedical Sciences, Queensland University of Technology, Brisbane 4001, Australia; ^4^​ School of Life and Environmental Sciences, The University of Sydney, Sydney 2006, Australia; ^5^​ Department of Infectious Diseases, John Hunter Hospital and the University of Newcastle, Newcastle 2305, Australia; ^6^​ Faculty of Science, Health, Education and Engineering, University of the Sunshine Coast, Sippy Downs 4072, Australia

**Keywords:** *Acinetobacter baumannii*, community-acquired pneumonia, whole-genome sequencing, epidemiology, virulence, antimicrobial resistance

## Abstract

*
Acinetobacter baumannii* causes severe, fulminant, community-acquired pneumonia (CAP) in tropical and subtropical regions. We compared the population structure, virulence and antimicrobial resistance determinants of northern Australian community-onset *A. baumannii* strains with local and global strains. We performed whole-genome sequencing on 55 clinical and five throat colonization *A. baumannii* isolates collected in northern Australia between 1994 and 2016. Clinical isolates included CAP (*n*=41), healthcare-associated pneumonia (*n*=7) and nosocomial bloodstream (*n*=7) isolates. We also included 93 publicly available international *A. baumannii* genome sequences in the analyses. Patients with *A. baumannii* CAP were almost all critically unwell; 82 % required intensive care unit admission and 18 % died during their inpatient stay. Whole-genome phylogenetic analysis demonstrated that community-onset strains were not phylogenetically distinct from nosocomial strains. Some non-multidrug-resistant local strains were closely related to multidrug-resistant strains from geographically distant locations. Pasteur sequence type (ST)10 was the dominant ST and accounted for 31/60 (52 %) northern Australian strains; the remainder belonged to a diverse range of STs. The most recent common ancestor for ST10 was estimated to have occurred in 1738 (95 % highest posterior density, 1626–1826), with evidence of multiple introduction events between Australia and Southeast Asia between then and the present day. Virulence genes associated with biofilm formation and the type 6 secretion system (T6SS) were absent in many strains, and were not associated with in-hospital mortality. All strains were susceptible to gentamicin and meropenem; none carried an AbaR resistance island. Our results suggest that international dissemination of *A. baumannii* is occurring in the community on a contemporary timescale. Genes associated with biofilm formation and the T6SS may not be required for survival in community niches. The relative contributions of host and bacterial factors to the clinical severity of community-onset *A. baumannii* infection require further investigation.

## Data Summary

Raw sequence data for northern and central Australian *A. baumannii* strains sequenced as part of this study are available in the Short Read Archive in Bioproject PRJNA478282 (https://www.ncbi.nlm.nih.gov/bioproject/?term=PRJNA478282) and accession numbers are listed in Table S1, available in the online version of this article. Accession numbers for global *A. baumannii* strains used in the analyses are listed in Table S2. GenBank accession numbers for annotated K loci are included in Table S3.

**Impact Statement**

Although best known for causing multidrug-resistant infection in hospitals, *Acinetobacter baumannii* is also an important cause of severe community-acquired pneumonia in tropical regions. This condition usually affects individuals with hazardous alcohol consumption, and often leads to death. In this study, we used whole-genome sequencing to investigate the genetic relationship between northern Australian community-onset *A. baumannii* strains and a collection of global strains. We also investigated the relationship between putative virulence genes and clinical outcomes. We found that some community-onset non-multidrug-resistant study strains were closely related to multidrug-resistant *A. baumannii* strains from geographically distant international locations, suggesting that global spread of *A. baumannii* is occurring in the community at the present day. We found variation in the profile of virulence determinants, although these were not associated with increased disease severity. We hypothesize that host factors play an important role in the susceptibility to and severity of community-onset *A. baumannii* infection.

## Introduction

*Acinetobacter baumannii* is a ubiquitous Gram-negative bacterium which is a frequent cause of multidrug-resistant nosocomial infection across the globe. The ability to survive for prolonged periods in the hospital environment and to readily acquire antimicrobial resistance determinants via horizontal gene transfer have contributed to the propensity of this organism to cause hospital outbreaks involving multiple facilities [1]. Three major international global clones have emerged as the dominant lineages associated with hospital outbreaks [2].

In contrast to nosocomial-acquired *A. baumannii *, little is known about community-acquired disease, which predominantly occurs in tropical and subtropical climates with a seasonal peak during the rainy season [3]. Unlike nosocomial infection, community-onset disease is not associated with multidrug resistance and usually affects individuals with risk factors such as hazardous alcohol consumption and diabetes [3, 4]. The clinical presentation is typically with fulminant lobar pneumonia with septic shock, with in-hospital mortality as high as 64 % [5].

The relative contributions of host and bacterial factors to the severity of community-onset *A. baumannii* pneumonia are uncertain. Virulence determinants in *A. baumannii* are incompletely understood, although biofilm formation, iron acquisition, lipo-oligosaccharide, capsular polysaccharide, outer membrane proteins and protein secretion systems are thought to be important [6]. Furthermore, alcohol intoxication may contribute to *A. baumannii* pathogenesis via impairment of phagocytosis and by influencing bacterial gene expression [5, 7–9].

To further our understanding of the genomic epidemiology and pathogenesis of community-onset *A. baumannii *, we used whole-genome sequencing to characterize the largest series of community-onset *A. baumannii* clinical isolates to date. We used comparative genomics to determine the phylogenetic relationship between these strains and a large collection of publicly available global *A. baumannii* strains, and to explore virulence determinants and genes associated with antimicrobial resistance.

## Methods

### Isolates and clinical definitions

Sixty *A. baumannii* strains isolated between 1994 and 2016 were sequenced, including 55 isolates from patients with clinical infection, and five throat colonization isolates from community subjects without clinical evidence of infection (Table S1) [3, 10]. All available clinical *A. baumannii* isolates were sequenced. All apart from three clinical isolates were collected from patients at Royal Darwin Hospital, which is the referral centre for the tropical north (‘Top End’) of the Northern Territory, Australia. The Top End has an area of roughly 245 000 km^2^ and is sparsely populated. Darwin is the only city and has a population of approximately 122 000 people; the remaining population lives in towns or remote communities separated by vast geographical distances. Three isolates were from Alice Springs Hospital, in the desert region of Central Australia. The five colonization isolates had been collected from patients presenting from the community to the Royal Darwin Hospital Emergency Department with non-respiratory presentations and a history of excess alcohol consumption [10].

Clinical isolates were from patients with community-acquired pneumonia (CAP; *n*=41), community-onset of pneumonia but recent healthcare contact (HCAP; *n*=7) or nosocomial bloodstream infection (BSI; *n*=7). Pneumonia was defined as clinical and radiological evidence of pneumonia [3]. For CAP and HCAP, *A. baumannii* was isolated within 48 h of hospital admission, and in nosocomial BSI ≥48 h after hospital admission [3, 11]. Only one isolate per episode of infection was included. Bloodstream isolates were the preferred sample type if available, and were considered significant if there was pure growth of *A. baumannii* in blood cultures and/or there was concurrent growth of *A. baumannii* in blood cultures and sputum. In the absence of a bloodstream isolate, sputum and pleural fluid isolates were considered significant if there was pneumonia and no alternative pathogen was identified during the episode of infection.

### Antimicrobial susceptibility testing, DNA extraction and whole genome sequencing

Antimicrobial susceptibility testing was performed on the Vitek 2 (bioMérieux) on all isolates except for two throat colonization isolates. Multidrug resistance was defined as resistance to two or more of the following drug classes: anti-pseudomonal cephalosporins, anti-pseudomonal carbapenems, fluoroquinolones and aminoglycosides [1]. Genomic DNA was extracted from the isolates using the DNeasy Blood and Tissue kit (Qiagen) according to the manufacturer’s instructions. Whole genome sequencing was performed at the Australian Genome Research Facility using Next-Era XT libraries and 100 bp paired-end reads on an Illumina HiSeq2500 sequencer. The raw sequence data are available in the Short Read Archive in Bioproject PRJNA478282.

### Bioinformatic analysis

Genomes were assembled using a reference-assisted approach with MGAP (v0.0.1) [12]. The Pasteur multilocus sequence type (MLST) of the study isolates was determined using the whole genome assemblies and https://pubmlst.org/abaumannii/. Genomes were annotated with Prokka 1.10 [13]. An additional 93 *A. baumannii* strains were obtained from public databases, selected to represent diverse geographical locations and sequence types (STs) (Table S2). Multiple sequence alignment and variant calling was undertaken using the SPANDx pipeline (v3.2.1) [14]. *A. baumannii* strain ATCC 17978 was used as the reference genome [15]. Phylogenetic analyses were performed using PAUP* [16] for maximum-parsimony and IQ-TREE [17] for maximum-likelihood analyses. For maximum-likelihood analyses, model selection was undertaken using the Bayesian information criterion and 1000 bootstrap replicates were performed to select a consensus tree. Adjustment of maximum-likelihood phylogenies for recombination was done using ClonalFrameML [18]. Temporal analysis was performed with BEAST [19] using the approach outlined by Holt *et al*. [20]. Phylogenetic trees were visualized using Interactive Tree of Life [21]. We determined the presence or absence of the *A. baumannii* virulence genes from the Virulence Factor Database [22], the *adeR/S* two-component regulatory system [23] and the type 6 secretion system (T6SS) cluster [24] in our collection using SRST2 [25] and Large Scale Blast Score Ratio (LS-BSR) [26]. Genes associated with antimicrobial resistance were identified using Abricate [27] and the NCBI Bacterial Antimicrobial Resistance Reference Gene Database version 1 [28].

## Results

### *A. baumannii* CAP usually requires intensive care unit admission and has a high mortality rate

The study included 55 clinical *A. baumannii* isolates; 41 were from patients with CAP, seven with HCAP and seven with nosocomial BSI including six with bacteraemic hospital-acquired pneumonia and one with catheter-associated bloodstream infection. The clinical isolates included one isolate per episode of infection, and included 51 bloodstream isolates. Three sputum isolates and a pleural fluid isolate from four patients with severe CAP requiring intensive care unit (ICU) admission were also included; these four patients had negative blood cultures and no alternative pathogens were identified. Clinical details for a subset of CAP and HCAP episodes have been previously reported [3]; the demographic and clinical features of all 55 patients with clinical *A. baumannii* infection included in this study are summarized in Table 1. All but three patients were Indigenous Australians. The number of cases of HCAP and nosocomial BSI in this study was small, but in all groups ≥50 % required ICU admission, and mortality was high and ranged from 14 to 29 %.

**Table 1.** Demographics and severity indicators for CAP, HCAP and nosocomial BSI episodes

	CAP (*n*=41)	HCAP (*n*=7)	Nosocomial BSI (*n*=7)	*P*-value
Median age (years), (range)	45.6 (29.8–68.8)	42.1 (31.1–48.5)	51.5 (41.2–76.1)	0.11
Male sex	18/39 (46 %)	4/7 (57 %)	2/7 (29 %)	0.55
Indigenous	33/36 (92 %)	7/7 (100 %)	6/6 (100 %)	1.0
Hazardous alcohol consumption	25/32 (78 %)	6/6 (100 %)	2/6 (33 %)	0.03
ICU admission	28/34 (82 %)	5/7 (71 %)	3/6 (50 %)	0.14
Inpatient death	7/40 (18 %)	1/7 (14 %)	2/7 (29 %)	0.84

### Clinical *A. baumannii* isolates comprise diverse and novel STs, but ST10 is the most common ST among CAP, HCAP and nosocomial strains

*In silico* MLST by the Pasteur scheme revealed that ST10 was the most common ST in this study, and accounted for 31/60 (52 %) isolates including 24/41 (59 %) CAP isolates, 4/7 (57 %) HCAP isolates and 3/7 (43 %) nosocomial BSI isolates (Table S1). Patient outcomes were similar in the ST10 and non-ST10-infected groups. ICU admission was required in 21/28 (75 %) ST10-infected patients versus 15/19 (79 %) infected with other STs, and in-hospital death occurred in 5/31 (16 %) and 5/23 (22 %), respectively (Table 2).

**Table 2.** Outcomes of *A. baumannii* infection in the presence and absence of putative virulence attributes Fisher's exact test *P-*values were >0.05 for all comparisons

Attribute (ST or virulence factor) present (+) or absent (–)		ICU admission	Died
ST10	+	21/28 (75 %)	5/31 (16 %)
	−	15/19 (79 %)	5/23 (22 %)
*hemO*	+	23/30 (77 %)	8/37 (22 %)
	−	13/17 (76 %)	2/17 (12 %)
*csuA-E*	+	14/17 (82 %)	4/20 (20 %)
	−	22/30 (73 %)	6/34 (18 %)
*abaI/R*	+	9/11 (82 %)	5/15 (33 %)
	−	27/36 (75 %)	5/39 (13 %)
*adeR/S*	+	15/19 (79 %)	4/22 (18 %)
	−	21/28 (75 %)	6/32 (19 %)
T6SS	+	18/24 (75 %)	7/30 (23 %)
	−	18/23 (78 %)	3/24 (13 %)

The remaining isolates were a diverse range of STs; these included ST216 (*n*=5) and ST267 (*n*=2), with the remainder being singletons including nine novel STs (Table S1). Only one isolate belonged to one of the major international global clones (GC1); this was a throat colonization isolate that was ST1. Both community and nosocomial strains were diverse, with 15 different STs among the 41 CAP isolates and five different STs among the seven nosocomial isolates.

### Whole-genome phylogenetic analysis shows that Australian community-onset and nosocomial strains are not unique, and are interspersed with global isolates

**Fig. 1.**
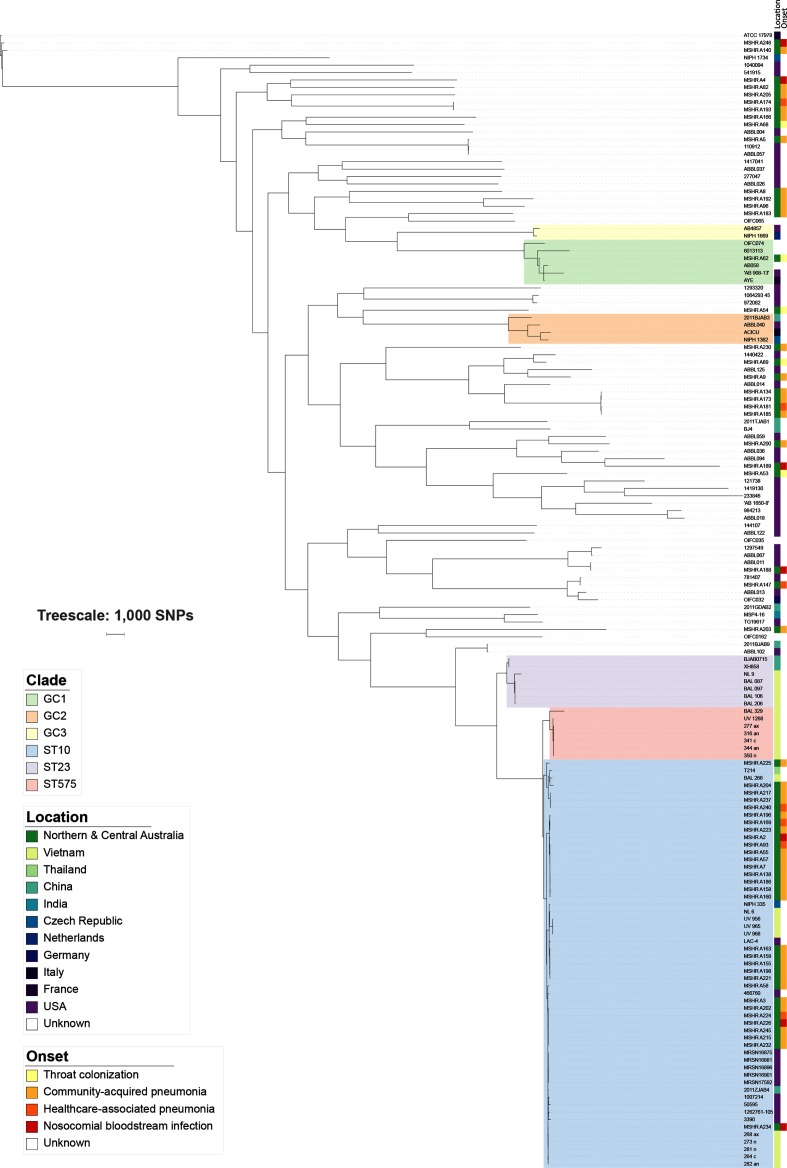
Maximum-parsimony phylogenetic analysis including Australian study and global *A. baumannii* isolates.

We undertook whole genome phylogenetic analysis of the 60 study isolates and an additional 93 international *A. baumannii* isolates selected for geographical and genetic diversity. The core alignment revealed 129 530 SNPs, which were used to inform a maximum-parsimony phylogeny (consistency index 0.2391, homoplasy index 0.7609; Fig. 1). Maximum-likelihood (Fig. S1) and recombination-adjusted (Fig. S2) analyses were also performed. In each of these analyses, the CAP, HCAP, nosocomial and colonization isolates were diverse and did not form distinct populations; some CAP strains were closely related to nosocomial BSI strains. Furthermore, the study isolates were spread among many different clades, and were interspersed with international isolates from diverse geographical locations throughout the phylogeny. There was no discernible phylogeographical signal.

### Australian non-multidrug-resistant ST10 strains are closely related to international multidrug-resistant ST10 strains

**Fig. 2.**
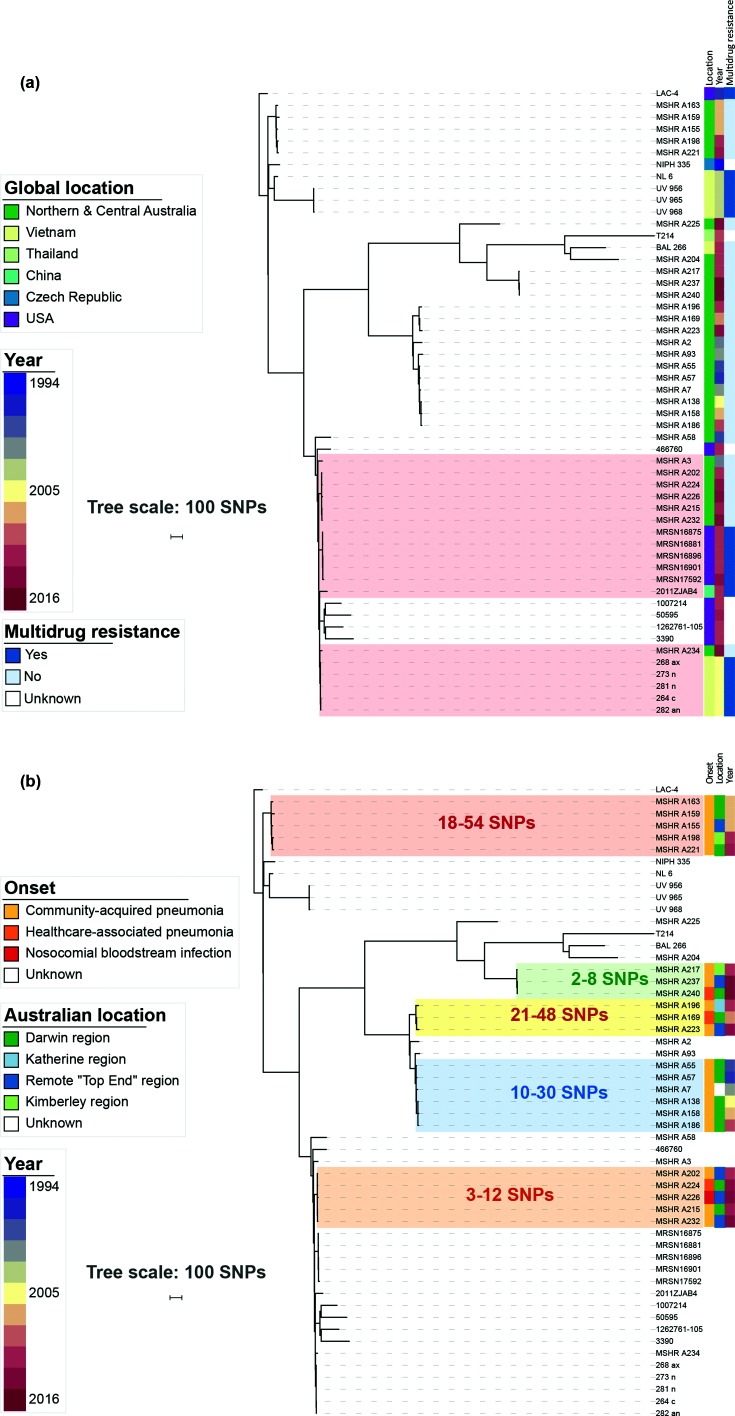
Maximum-parsimony phylogenetic analysis of ST10 *A. baumannii* strains. (a) International and Australian isolates separated by <100 SNPs are highlighted. (b) Local Australian genomic clusters are highlighted, and the range of SNP distances between isolates in each cluster is listed.

**Fig. 3.**
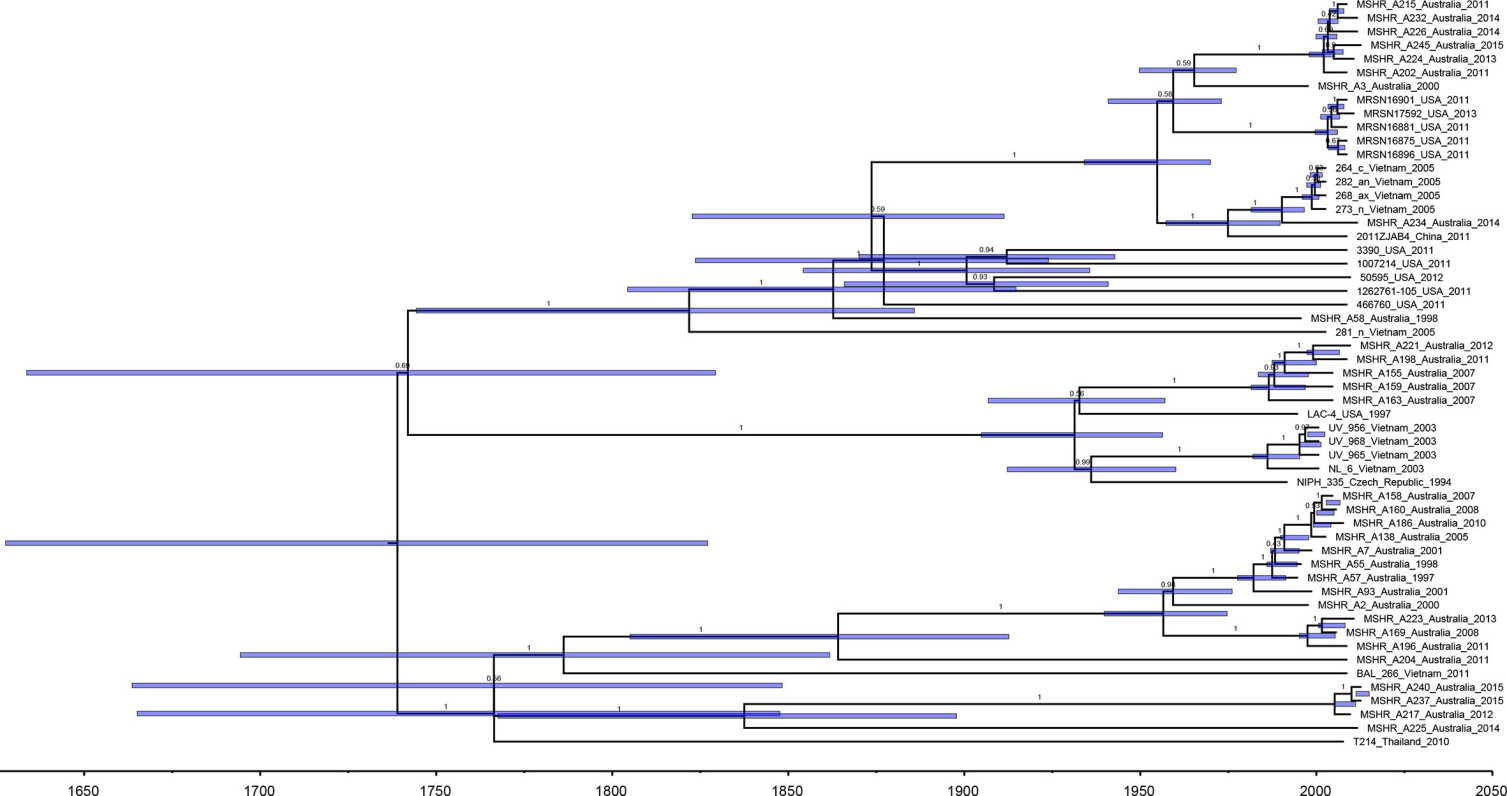
Bayesian phylogenetic analysis of ST10 *A. baumannii* strains. Blue bars indicate 95% HPD. Branch labels indicate posterior probabilities. Timescale indicated in years.

ST10 strains formed a single clade including several smaller clusters of closely related isolates. To examine this further, local and international ST10 sequences were mapped against the ST10 LAC-4 genome [29] resulting in a maximum-parsimony phylogeny based on 6560 core SNPs (Fig. 2). Maximum-likelihood and recombination-adjusted ST10 phylogenies are presented in Figs S3 and S4. A dated ST10 phylogeny is shown in Fig. 3. The most recent common ancestor (MRCA) for ST10 was estimated to date back to 1738 (95 % highest posterior density, HPD, 1626–1826). There were three deeply branching clades, each including strains from northern Australia and Southeast Asia, suggesting multiple introduction events between then and the present day.

There were several instances where our non-multidrug-resistant study strains were closely related to multidrug-resistant strains from geographically distant locations including Vietnam, the USA and China (Figs 2a and 3). A 2014 nosocomial BSI isolate from our study was separated from a cluster of colonization isolates collected from patients entering an ICU in Vietnam in 2005 [30] by 22 core SNPs, and was separated by 83 core SNPs from a 2011 carbapenem-resistant bloodstream isolate from Zhejiang, China (2011ZJAB4) [31]. Six community and nosocomial isolates from our study collected between 2011 and 2015 were separated from a clade of 2011 multidrug-resistant outbreak strains from Maryland, USA [32] (labelled ‘MRSN’ in the tree), by 59 core SNPs. The patients infected with these strains had no known epidemiological links to these international locations, and some of the patients were from very remote locations in the Top End. The MRCA for this clade was estimated to have occurred in 1957 (95 % HPD 1936–1972) (Fig. 3), the approximate time that both Australia and the USA were participating in the Vietnam war.

Also within the ST10 tree were five distinct genomic clusters of closely related study strains (Fig. 2b). Each of these clusters comprised community-onset strains; the clusters spanned 4–14 years with 4/5 clusters involving multiple northern Australian regions separated by hundreds of kilometres. Despite distances of as little as two SNPs separating some strains, the local genomic clusters each spanned a long time period and/or broad geographical space, and there were no clear epidemiological links between patients. The MRCAs for each of these clusters were estimated to have occurred between 7 and 22 years prior to the earliest strain in each cluster.

### Putative virulence genes are absent in many northern Australian strains

**Fig. 4.**
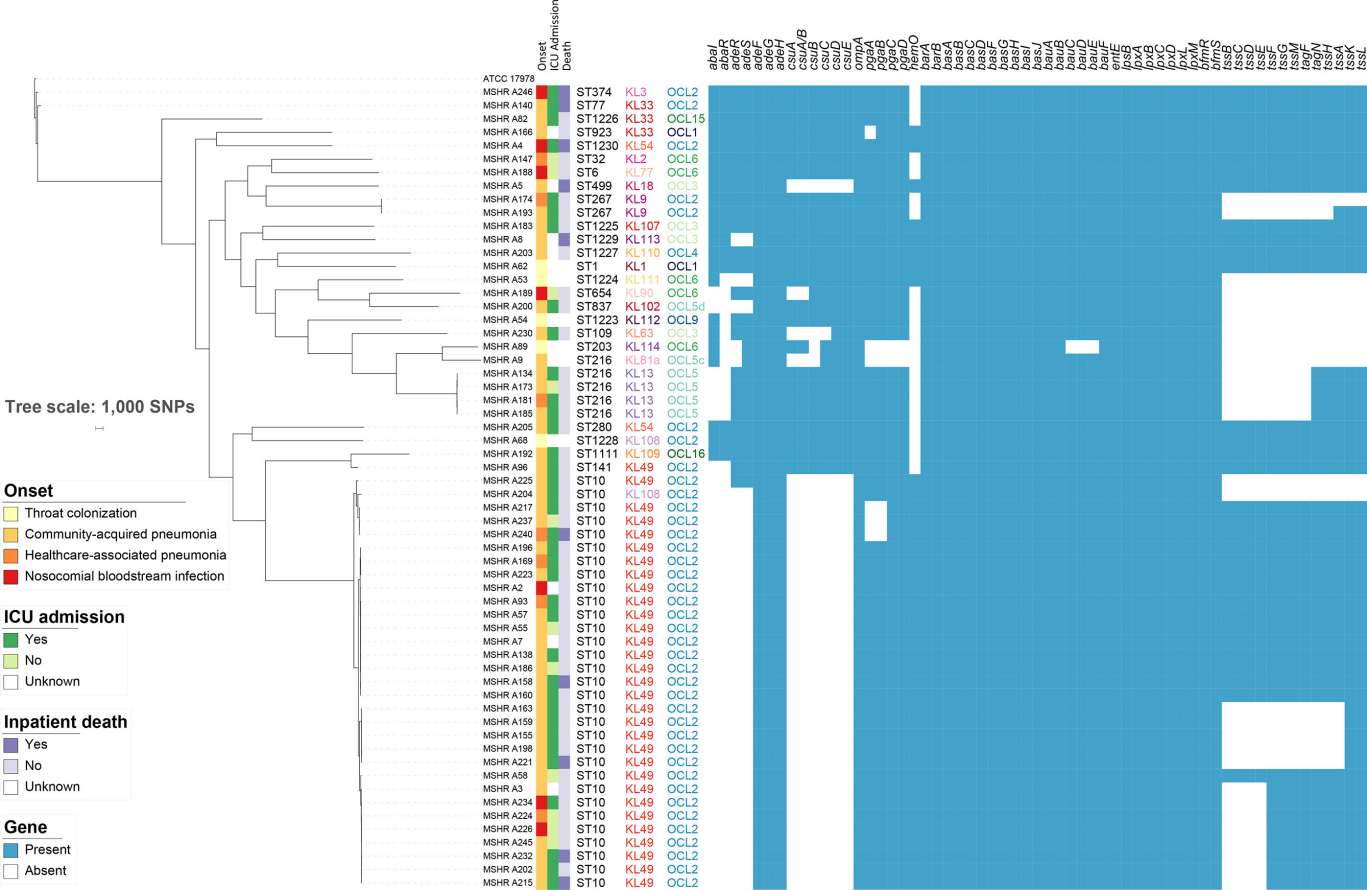
Virulence genes, and onset and outcomes of *A. baumannii* infection.

Interrogation of the study and international genomes for the presence of putative virulence genes revealed an absence of several virulence genes. The presence or absence of virulence genes varied according to lineage but not disease onset (Fig. 4). Associated with biofilm formation, the chaperone-usher type 1 pili system is encoded by the *csuAB-E* gene cluster [33]; 36/60 (60 %) study isolates did not have a complete *csuAB-E* cluster, and the locus was entirely absent in all ST10 strains. The *abaI* and *abaR* autoinducer synthase genes [34] are associated with quorum sensing and biofilm formation; one or both of these genes were absent in 43/60 (72 %) study isolates, and both were absent in all ST10 strains. The two-component regulatory system *adeRS* regulates expression of *adeABC* and is associated with increased biofilm formation [23]; one or both of these genes were absent in 35/60 (58 %) study strains and both were absent in all ST10 strains except one. T6SS genes were also absent in some isolates; the locus was either partly or completely absent in 28/60 (47 %) study isolates including 15/31 (48 %) ST10 strains. A haem utilization cluster including the *hemO* gene was previously found to be present in hypervirulent ST10 strains [29] and variably present in other *A. baumannii* genomes. The *hemO* gene was found in 39/60 (65 %) strains in our study, including all ST10 strains.

The proportion of patients infected with strains with and without these virulence factors requiring ICU admission and who died during their hospital admission is presented in Table 2. None of these virulence factors demonstrated a statistically significant association with ICU admission or inpatient death (Fisher's exact test *P*>0.05 for all comparisons), although the number of cases was small.

### Northern Australian *A. baumannii* strains have diverse capsule K loci and lipo-oligosaccharide OC loci

In *A. baumannii *, the polysaccharide capsule which forms a protective layer on the cell surface is considered a major virulence determinant [35, 36]. The enzymes required for capsule biosynthesis are encoded at the K locus, but there is extensive variation at this site leading to differences in the composition and structure of the capsule [37]. Consistent with the diversity of the isolates in this collection, the study isolates had 22 different capsule types (Fig. 4, Table S1) including eight novel capsule types designated KL108–114; annotated sequences for these have been uploaded to GenBank, and accession numbers are listed in Table S3. All ST10 strains with the exception of one had the KL49 capsule locus associated with the production of 8-epilegionaminic acid. The remaining ST10 isolate carried KL108, which carries genes for legionaminic acid synthesis. The isolate carrying KL108 was closely related to the remaining local ST10 isolates, suggesting the replacement event had occurred recently and locally. Four isolates belonging to ST216 had the KL13 locus, which is associated with the production of acinetaminic acid, a recently described non-2-ulosonic acid that had not previously been identified in a natural biological source [38]. The outer core of lipo-oligosaccharide also varies significantly due variation in gene sets at the OC locus responsible for directing synthesis [39], and several different OC loci were also seen in the collection, including four that had not been detected previously (OCL13–16) (Fig. 4, Table S1).

### Community-onset strains are not multidrug-resistant and do not harbour an AbaR resistance island

**Fig. 5.**
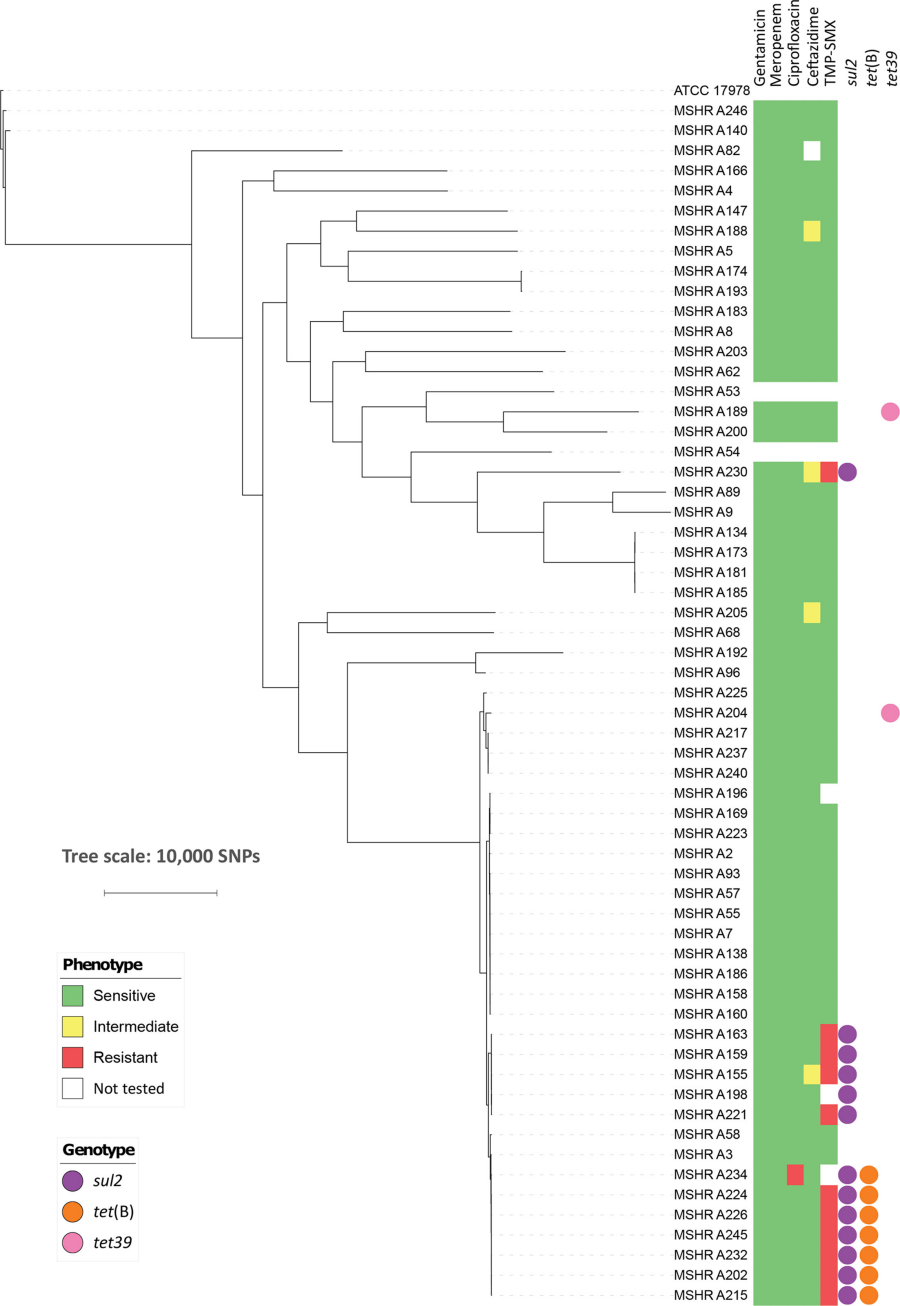
Phenotypic antimicrobial susceptibilities and antimicrobial resistance genes.

Antimicrobial susceptibility results were available for all isolates apart from two throat colonization isolates (Fig. 5). All isolates tested were susceptible to gentamicin and meropenem. One isolate was resistant to ciprofloxacin, four had intermediate susceptibility to ceftazidime, and 13 were resistant to trimethoprim-sulfamethoxazole (TMP-SMX). The *comM* gene is the usual site of insertion for AbaR resistance islands in GC1 and GC2 *A. baumannii* isolates, and was intact in 51/60 isolates. The nine strains with genes inserted within *comM* did not have genes associated with antimicrobial resistance at this site; instead, a wide variety of genes associated with metabolism, transcriptional regulation and heavy metal transport were identified.

The one ciprofloxacin-resistant isolate (MSHR_A234) was nosocomial, and had an S83L substitution in *gyrA* and an S80L substitution in *parC*. This combination of mutations is common in *A. baumannii *, is associated with high-level resistance to quinolones [40, 41] and was the probable cause of ciprofloxacin resistance in this strain. Isolates with phenotypic resistance to TMP-SMX all had the *sul2* gene, known to be associated with resistance to sulfamethoxazole in Gram-negative bacteria [42]. In total, 10/11 of these isolates were ST10 strains. Seven of the TMP-SMX-resistant isolates also possessed *tet*(B) and an additional two isolates had the *tet39* gene; these genes are associated with resistance to tetracyclines [43]. Phenotypic testing was not undertaken for this class of antimicrobial because it is not used for treatment of *A. baumannii* infection at our institution, and the clinical significance of the *tet* genes is uncertain.

## Discussion

In this study, we used comparative genomics to characterize the largest reported collection of community-onset *A. baumannii* isolates. We found that northern Australian community-onset isolates were diverse, were not phylogenetically distinct from international isolates and in some cases were closely related to multidrug-resistant nosocomial strains from geographically distant locations [44]. We found no evidence of a difference in the presence of putative virulence genes in community-onset isolates. These findings suggest that the typical presentation of severe, rapidly progressing community-acquired *Acinetobacter* pneumonia may relate more to known host risk factors than to genetic variants in the infecting pathogen. There may be important as yet undiscovered virulence determinants among these strains, and a genome-wide association study using a larger and more diverse dataset could be used to uncover these. Another possible explanation is that acquisition of genes associated with antimicrobial resistance may have an attenuating effect on the virulence of nosocomial *Acinetobacter* strains, leading to a more indolent presentation.

The lack of any discernible geographical signal and the close evolutionary relationships between strains from geographically distant locations suggests that international dissemination of *A. baumannii* is occurring on a contemporary timescale. Temporal analysis revealed that the MRCA for ST10 was estimated to have occurred in approximately 1738, with multiple introduction events between northern Australia and Asia between then and the present day. This timescale is longer than for the emergent, multidrug-resistant GC1 lineage, which was estimated to have an MRCA as recently as 1960 [20]. Outside the healthcare setting, *Acinetobacter* species are generally considered to be environmental micro-organisms and *A. baumannii* has previously been isolated from soil and water in tropical and temperate climates [45–48]. *A. baumannii* also has community niches outside the natural environment, and colonizes humans, their lice, domestic and farm animals, and a wide variety of foods including fruit and vegetables, cheese, meat and milk [10, 45–47]. Each of these is a potential vessel for dissemination, and may contribute to spread of *A. baumannii* in the community, conceivably over large geographical distances.

Additionally, *A. baumannii* infection was described as a complication of trauma during the Vietnam war [49], more recently in the Middle East [50] and as a result of the 2006 conflict in East Timor [51], and has also occurred following natural disasters including the 2004 Asian tsunami [52]. Whether infecting strains are acquired from the individual’s microbiome, the local environment or from healthcare facilities is uncertain. Our temporal analyses suggested that the MRCA for a clade of closely related ST10 strains from northern Australia, Vietnam and USA was estimated to have occurred in approximately 1957 and we hypothesize that ST10 *A. baumannii* dissemination may have occurred as a result of repatriation of trauma victims during the Vietnam war.

ST10 was the most common ST seen among community and nosocomial isolates in this study. Given that the same clone dominated in both settings, we hypothesize that patients with nosocomial infection may have already been colonized at the time of hospital admission prior to developing infection with these strains. This is analogous to infection with methicillin-resistant *Staphylococcus aureus *, in which genomic sequencing has demonstrated intermingling of community and nosocomial strains [53]. It has previously been suggested on the basis of PFGE of community colonization and hospital *A. baumannii* strains in New York that the community is not a reservoir for hospital *A. baumannii* strains [5, 48], although our findings suggest this may not be the case in our region. This suggestion is also challenged by findings of a study using the Oxford MLST scheme in Taiwan, which found that half of the dominant nosocomial STs also caused community-onset disease [54].

Internationally, ST10 strains have been associated with colonization and nosocomial infection in an ICU in Vietnam [30], and an outbreak of extensively drug-resistant infection in the USA [32], suggesting that this ST exists in diverse geographical locations and has the capacity to colonize, cause disease and to acquire antimicrobial resistance determinants. Furthermore, it has previously been suggested that ST10 strains are more virulent than others; evidence to support this has included the high mortality rate associated with an outbreak in the USA among ‘relatively immunocompetent’ individuals [32], and demonstration of hypervirulence of ST10 strains in mouse models of infection [32, 55]. The majority of patients included in our study had severe pneumonia requiring ICU admission; however, within our cohort, ST10 strains were not associated with increased ICU admission or inpatient death.

The remaining community and nosocomial isolates in our study belonged to a wide range of diverse STs. This is consistent with the extensive ST diversity seen among both community and nosocomial strains in Taiwan, with unique STs found in 78 and 31 %, respectively [54]. There was no dominant community ST in Taiwan, but 17 % of community-onset isolates were Oxford ST447, an ST found in 27/31 (87 %) of our Pasteur ST10 strains including community-onset and nosocomial strains. Extensive ST diversity has also been observed in community *A. baumannii* strains outside the human clinical setting. A survey of soil, water, food, and domestic and farm animals in Lebanon identified 42 *A. baumannii* isolates belonging to 36 STs, including 24 novel STs [47]. These included two STs observed in our study, including an ST10 strain isolated from cow faeces and an ST216 strain isolated from cheese. In Reunion Island, 12 isolates from dogs and cats belonged to eight STs, and included one ST observed in our study; this was an ST203 strain isolated from a cat [46]. These observations highlight that the *A. baumannii* STs that cause severe disease may have diverse niches in wide-ranging geographical locations.

We observed that genes associated with biofilm formation and the T6SS varied according to lineage, potentially reflecting adaptation to different ecological and environmental conditions. The absence of T6SS genes and *csuAB-E* in clinical ST10 strains has been previously described [32, 56] and we observed the absence of these genes in addition to the absence of *abaI/R* and *adeR/S* in ST10 strains in our collection, despite the severity of clinical disease. The latter three loci are associated with biofilm formation and may be more important for persistence in the hospital environment than in other niches. This may explain why, although capable of causing nosocomial outbreaks [30, 32], ST10 is not a common cause of nosocomial *A. baumannii* infection globally. T6SS is thought to have a role in host colonization and killing of competing bacteria, and in *Acinetobacter* is hypothesized to have a role in the release and uptake of the DNA belonging to bacterial prey, potentially contributing to acquisition of antimicrobial resistance genes [57]. This may also provide a greater advantage for nosocomial compared to community strains.

The clinical significance of variation at the K locus is uncertain. The only previous study to assess the effect on virulence investigated the KL1 type and found that this was associated with increased survival in human ascites fluid and human serum, and increased virulence in a rat soft tissue model compared to KL1-negative strains [36]. The KL49 locus is associated with the production of 8-epilegionaminic acid, and has previously been found in the hypervirulent ST10 LAC-4 strain [58], the ST10 isolates associated with a fatal outbreak in the USA [32] and almost all ST10 strains in our collection. Recently, Oxford ST457 strains carrying KL49 were found to be associated with greater mortality and were more virulent in a *Galleria mellonella* infection model than other CC92 strains [59]. Whether the KL49 capsule type may contribute to increased virulence requires further investigation.

The main limitation of this study was the small sample size. We hypothesize that there are important yet-to-be discovered virulence factors that contributed to the severity of illness observed in our study. These could be identified using a genome-wide association study (GWAS) comparing various clinical outcomes, or comparing disease with colonization. Within our cohort of clinical cases, there was little heterogeneity in terms of disease severity, i.e. almost everyone was critically unwell. Additionally, there were only five colonization isolates. This led to inadequate power to perform GWAS using our current dataset. While there are a large number of publicly available *A. baumannii* genomes, there are few associated data relating to clinical outcomes, and data related to invasive disease versus colonization may be unreliable due to the clinical complexity of making this distinction. We therefore did not proceed with GWAS including publicly available strains as part of the current study.

Here, we have described the population structure, virulence and antimicrobial resistance determinants in a collection of predominantly community-onset *A. baumannii* strains from northern and central Australia. We found that ST10 was the dominant ST, and was a cause of CAP, HCAP and nosocomial *A. baumannii* infection in our setting. The remaining isolates were very diverse, and only one isolate belonged to one of the major international global clones. We found no evidence of phylogeographical restriction, and in some cases non-multidrug-resistant community-onset strains were closely related to multidrug-resistant strains from geographically distant locations. We hypothesize that dissemination may have occurred via humans, ectoparasites, food and/or inanimate objects. Virulence genes associated with biofilm formation and the T6SS were absent from many of our isolates, which suggests these attributes may not be required for survival in community niches. Although there was variation in the virulence gene profile of *A. baumannii* strains in this collection, pathogen factors contributing to the typical presentation of acute, fulminant pneumonia are uncertain. Key host risk factors such as hazardous alcohol intake and diabetes may play a greater role than variations in bacterial virulence genes in the pathogenesis of severe *A. baumannii* pneumonia, and further work investigating host–pathogen interactions is required.

## Data Bibliography

1. Raw sequence data for northern and central Australian *A. baumannii* strains sequenced as part of this study are available in the Short Read Archive in Bioproject PRJNA478282 (https://www.ncbi.nlm.nih.gov/bioproject/?term=PRJNA478282) and accession numbers are listed in Table S1. References and accession numbers for global *A. baumannii* strains used in the analyses are listed in Table S2.

## Supplementary Data

Supplementary File 1

Supplementary File 2
