# Supplementary material for: Genomic epidemiology of severe community-onset Acinetobacter baumannii infection
Source: Microb Genom. 2019 Feb 26;5(3):e000258. doi: 10.1099/mgen.0.000258 (PMC6487312; doi:10.1099/mgen.0.000258)
Supplement: Supplementary File 1 [file mgen-5-258-s001.pdf]

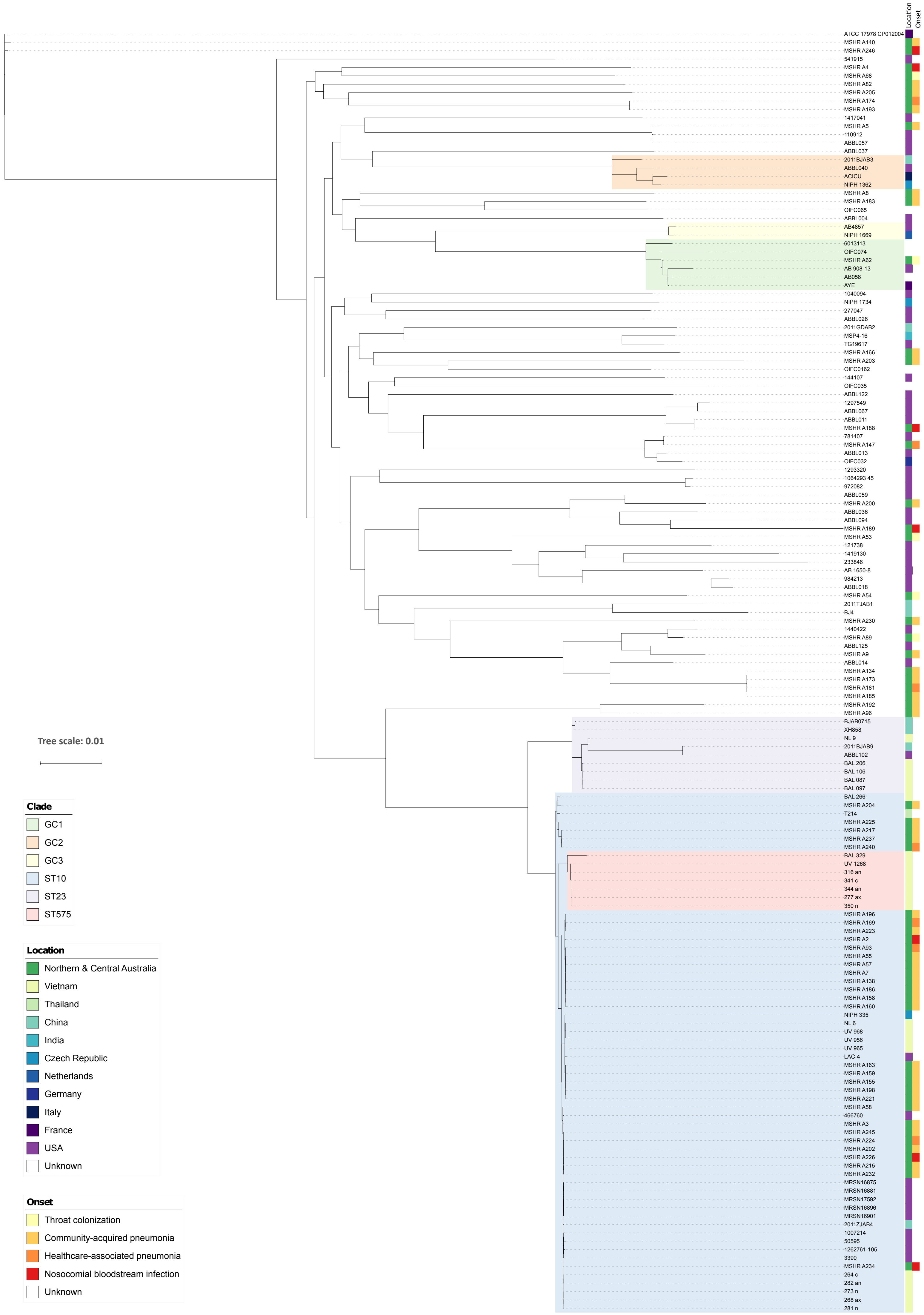

Figure S1. Maximum-likelihood global *A. baumannii* phylogeny. Scale bar indicates number of substitutions per site.

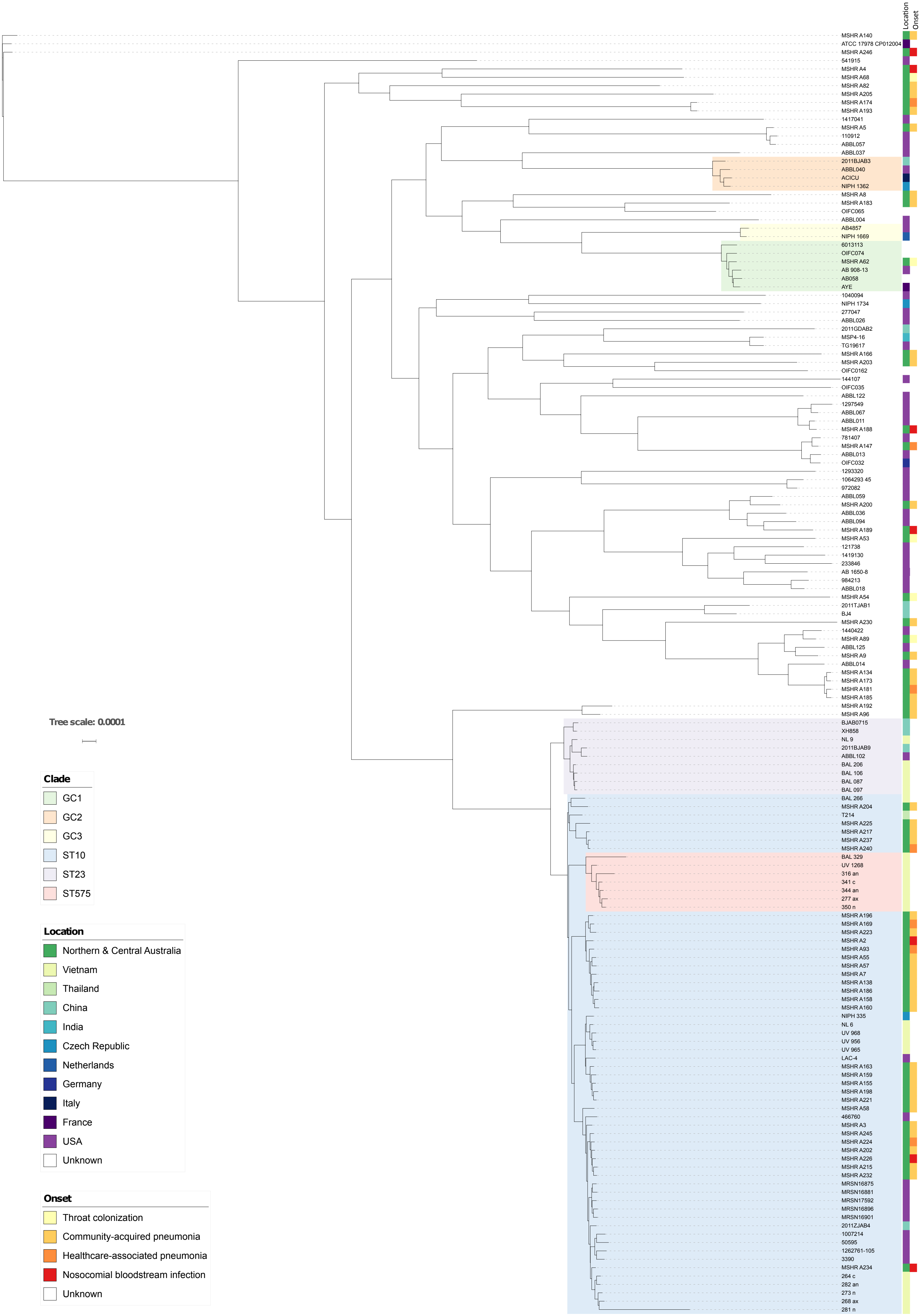

Figure S2. Recombination-adjusted global *A. baumannii* phylogeny. Scale bar indicates number of substitutions per site.

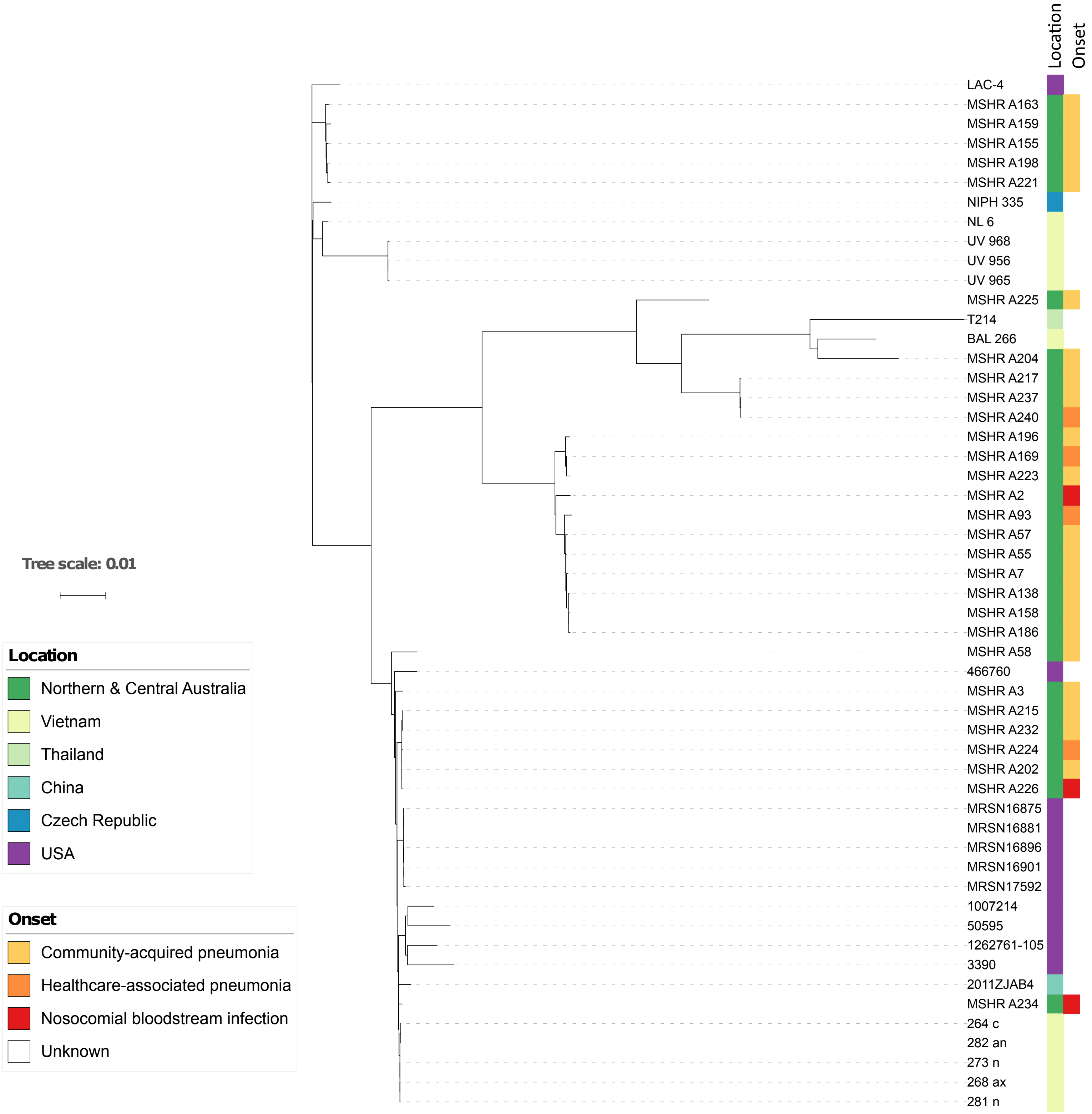

Figure S3. Maximum-likelihood *A. baumannii* ST10 phylogeny. Scale bar indicates number of substitutions per site.

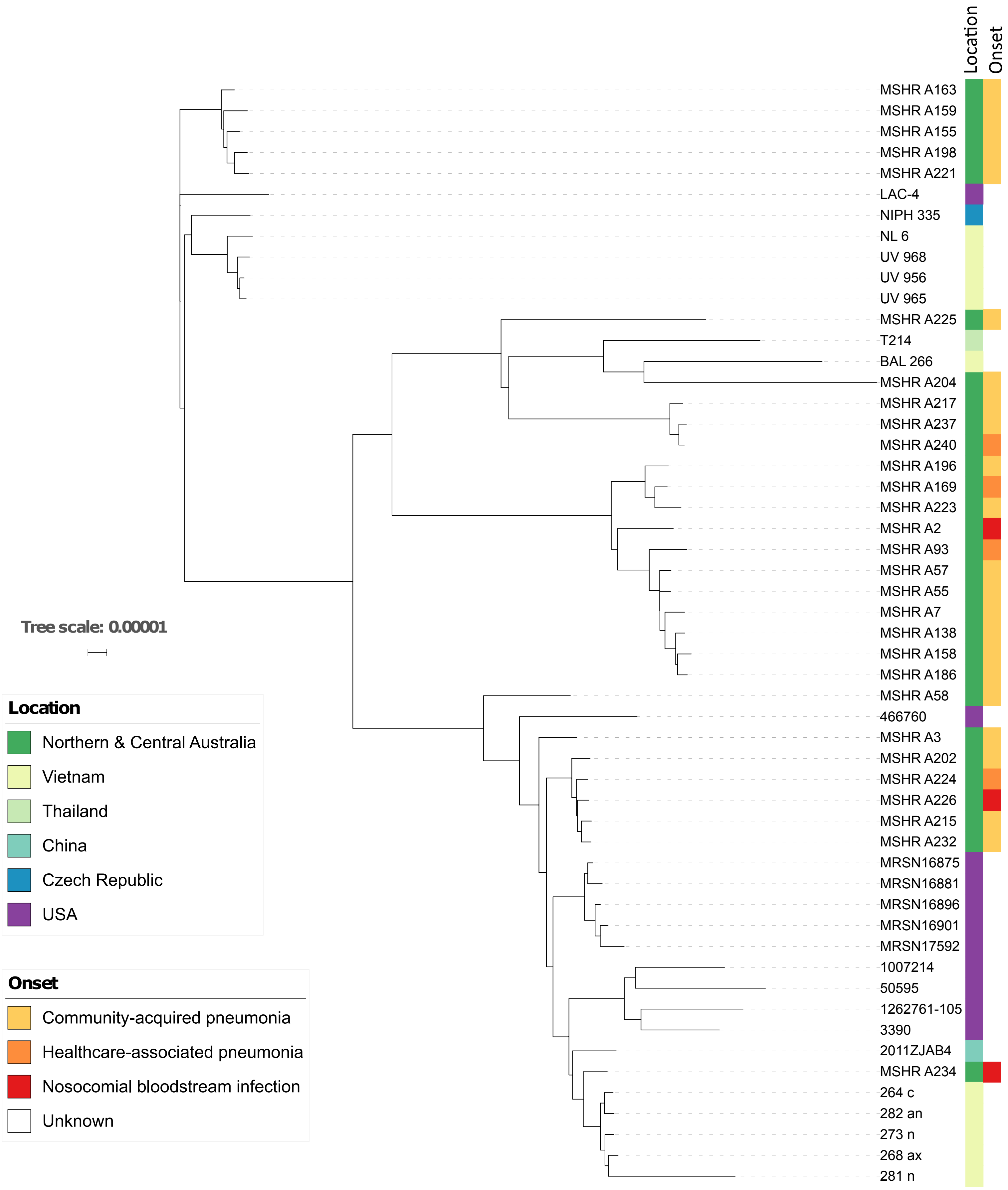

Figure S4. Recombination-adjusted *A. baumannii* ST10 phylogeny. Scale bar indicates number of substitutions per site.
